# Extensive monitoring of the natural menstrual cycle using the serum biomarkers estradiol, luteinizing hormone and progesterone

**DOI:** 10.1016/j.plabm.2021.e00211

**Published:** 2021-03-13

**Authors:** Ellen Anckaert, Alexander Jank, Julia Petzold, Felix Rohsmann, Rhonda Paris, Martin Renggli, Kathrin Schönfeld, Johan Schiettecatte, Monika Kriner

**Affiliations:** a Laboratory of Hormonology and Tumour Markers, University Hospital Brussels (UZ Brussel), Free University of Brussels, Laarbeeklaan 101, B-1090, Brussels, Belgium; b University Hospital Leipzig, Liebigstraße 20, 04103, Leipzig, Germany; c Praxis Dr Felix Rohsmann, Sindelsdorfer Str. 12, 82392, Habach, Germany; d Roche Diagnostics, 9115 Hague Rd, 46256, Indianapolis, IN, USA; e Trina Bioreactives AG, Grabenstrasse 8, 8606, Nänikon, Switzerland; f Roche Diagnostics GmbH, Nonnenwald 2, 82377, Penzberg, Germany; g Triga-S, Mühltal 5, 82392, Habach, Germany

**Keywords:** Luteinizing hormone, Estradiol, Progesterone, Normo-ovulatory, Natural menstrual cycle, CI, confidence interval, E2, estradiol, Gen III, third-generation, LH, luteinizing hormone, IQR, interquartile range

## Abstract

Expected values for estradiol (E2), luteinizing hormone (LH), and progesterone determined in serum allow accurate assessment of menstrual cycle phase. Automated immunoassays demonstrate variable degrees of bias, emphasizing the need to establish method-specific reference values. We therefore established method-specific reference intervals for the Elecsys® LH assay and new generation Elecsys Estradiol III and Progesterone III assays (cobas e 801 analyzer) in 85 apparently healthy women aged 22–37 (US)/18–37 (EU) years over one natural menstrual cycle. Cycle length and day of ovulation were standardized; phases were defined by LH surge and/or progesterone/E2 levels. Median (5th–95th percentile) concentrations (follicular/ovulation/luteal) were E2: 198 ​pmol/L (114–332), 757 ​pmol/L (222–1959) and 412 ​pmol/L (222–854); LH: 7.14 IU/L (4.78–13.2), 22.6 IU/L (8.11–72.7) and 6.24 IU/L (2.73–13.1); progesterone: 0.212 ​nmol/L (0.159–0.616), 1.81 ​nmol/L (0.175–13.2) and 28.8 ​nmol/L (13.1–46.3). Sub-phase (early/intermediate/late) reference values were also determined for follicular and luteal phases. This multicenter study established reliable, method-specific E2, LH and progesterone reference values that could assist clinical decision-making in women with fertility disorders and monitoring of natural cycles in assisted reproductive treatment.

## Highlights

• E2, LH and progesterone are biomarkers for assessing natural menstrual cycle phase.
• These biomarkers support diagnosis, monitoring and treatment of fertility disorders.
• E2, LH and progesterone profiles were derived using new generation Elecsys® assays.
• Expected serum values are presented for each menstrual cycle phase and sub-phase.
• Data will inform clinical decision-making for women with fertility disorders.

## 1 Introduction

Estradiol (E2), luteinizing hormone (LH) and progesterone are established biomarkers determined in serum to characterize the natural menstrual cycle, each following a cyclical pattern tightly controlled by the hypothalamic-pituitary-gonadal axis [1]. Based on their role in regulating the menstrual cycle, measurement of these hormones can facilitate diagnosis, monitoring and treatment of a range of medical conditions, including polycystic ovary syndrome and hormone-related infertility [2].

Substantial inter-individual and inter-cycle variation has been observed in serum profiles for E2, LH and progesterone [[3], [4], [5]], particularly in time of onset, amplitude and duration of the LH surge associated with ovulation [4]. Expected values for E2, LH and progesterone have previously been determined in urine [3,6]. However, these values may not accurately reflect serum profiles [6], which may provide a more accurate and reliable means of classifying menstrual cycle phase and/or sub-phase (i.e. early/intermediate/late follicular and luteal phases) than those determined in urine. Furthermore, automated immunoassays for E2 and progesterone have previously demonstrated variable degrees of bias [7,8]. Detailed, method-specific serum hormone profiles could help to inform clinical decision-making for normo-ovulatory women attempting to conceive naturally or for women with infertility issues undergoing counseling for assisted reproductive therapy [9].

We aimed to determine method-specific expected values for serum E2, LH and progesterone, throughout the natural menstrual cycle in normo-ovulatory women, using the Elecsys® LH [10] and the new generation Elecsys Estradiol III [11] and Elecsys Progesterone III [12] electrochemiluminescence immunoassays.

## 2 Materials and methods

In addition to the following methodology, further details are provided in the Supplementary materials (e.g. eligibility criteria, data collection, sample storage, menstrual cycle standardization and statistical analyses).

### 2.1 Study design

This multicenter, prospective, non-interventional study enrolled participants at two sites in the USA (Roche Wellness Center Indianapolis; Trina Bioreactives AG [Contract Research Organization]) and three sites in Europe (UZ Brussels; Universitätsklinikum Leipzig; Praxis Dr Felix Rohsmann) between June 2016 and August 2017. The study was conducted in accordance with the principles of the Declaration of Helsinki and the International Council for Harmonization Guidelines for Good Clinical Practice. All women provided signed, written informed consent. The study protocol was approved by institutional review boards prior to study initiation.

### 2.2 Participants

Apparently healthy women aged 22–37 years (US) or 18–37 years (EU) with a natural menstrual cycle (24–35 days; confirmed by a physician) were enrolled. General health was assessed via a validated questionnaire prior to enrollment.

### 2.3 Sample collection

Blood samples (10 ​mL whole blood per venipuncture) were obtained from each participant approximately three times per week for the duration of one menstrual cycle (24–35 days; 7–15 samples in total) between two consecutive menstrual bleedings.

### 2.4 Determination of E2, LH and progesterone values

Values were determined for E2, LH and progesterone using the Elecsys Estradiol III, Elecsys LH and Elecsys Progesterone III immunoassays (Roche Diagnostics International Ltd, Rotkreuz, Switzerland) on the cobas e 801 analyzer. All assays were used according to the manufacturer’s instructions. Further detail on these immunoassays is provided in the Supplementary methods section.

All participants’ hormone profiles across the menstrual cycle were evaluated by a physician. Menstrual cycle length and day of ovulation were standardized prior to determination of biomarker serum levels to account for variance in cycle lengths (24–35 days), and to enable determination of values during additional cycle sub-phases. Following standardization, the cycle length for all samples was 29 days, with LH peak occurring at day 15 (i.e. the day of ovulation). Further detail on standardization of cycle length and day of ovulation is provided in the Supplementary methods section.

## 3 Results

### 3.1 Analysis population

A total of 208 participants were enrolled, of which 85 met the eligibility criteria. The most common reason for exclusion was no evidence of an LH peak and/or low progesterone levels at the mid-luteal phase, indicative of deficient corpus luteum function **(**Supplementary Fig. S1). Participants had a median age of 25 years and median body mass index of 23 ​kg/m^2^ (Supplementary table S1). The majority of participants were white/Caucasian (82.4%) and 7.06% worked nightshifts ≥3 times per week.

### 3.2 Determination of E2, LH and progesterone values for menstrual cycle phases

**Fig. 1:**
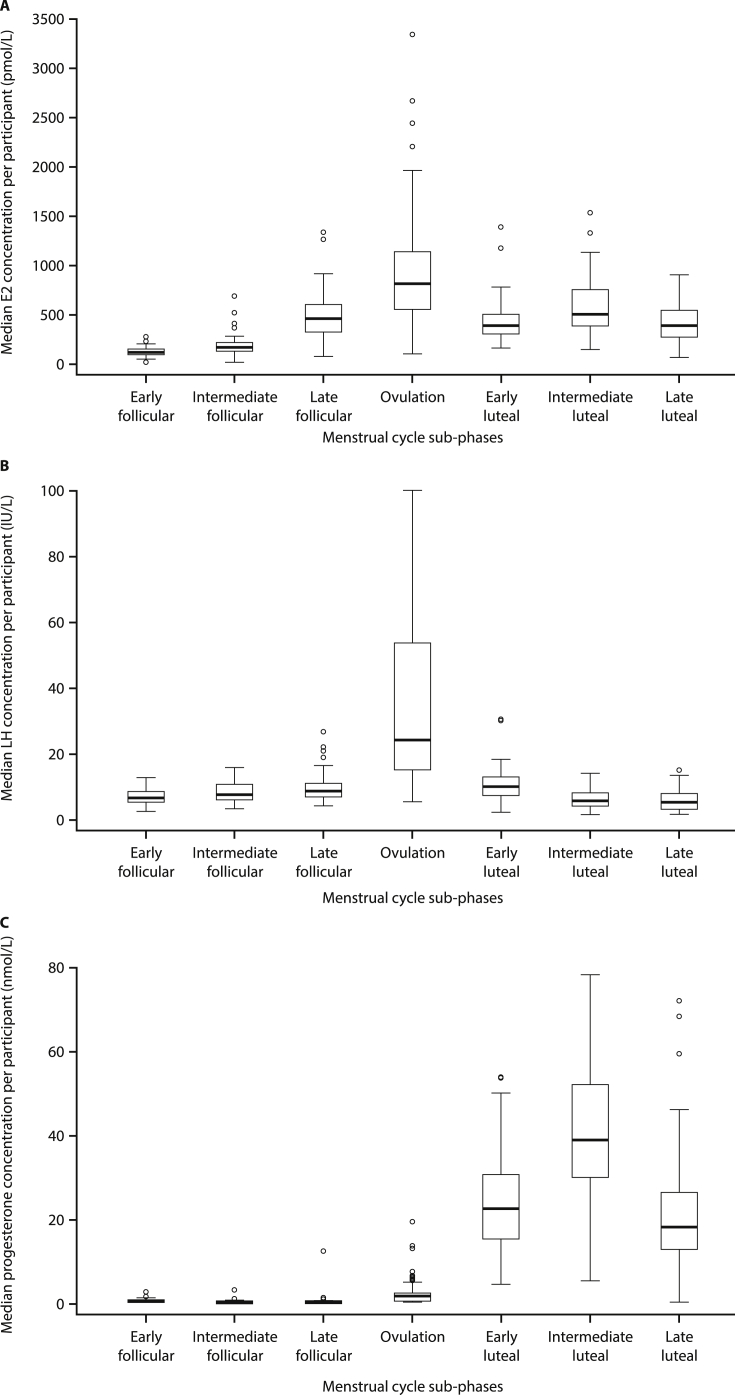
Boxplots of median values for (A) E2 (pmol/L), (B) LH (IU/L) and (C) progesterone (nmol/L) per participant, by menstrual cycle sub-phase. E2, estradiol; LH, luteinizing hormone.

Median values for E2, LH and progesterone are presented by menstrual cycle phase (follicular, ovulation and luteal phase) in Table 1 and Fig. 1. Median E2 concentrations were highest during ovulation (757 ​pmol/L) and also showed the greatest interquartile range (IQR) during this phase. Median LH values peaked during the ovulation phase (22.6 IU/L). Median progesterone concentrations increased over the course of the menstrual cycle and were highest during the luteal phase (28.8 ​nmol/L).

**Table 1:** Summary of median concentrations and 5th–95th percentiles for E2, LH and progesterone, for the three main phases and seven sub-phases of the menstrual cycle.

Menstrual cycle phase		*N*^a^	Analyte concentration		
			Median	5th percentile (90% CI)	95th percentile (90% CI)
Analysis of main menstrual cycle phases: E2 concentration (pmol/L)^b^					
Follicular		85	198	114 (19.1–135)	332 (322–637)
Ovulation		81	757	222 (98.5–283)	1959 (1598–3338)
Luteal		85	412	222 (159–280)	854 (760–1334)
Analysis of menstrual cycle sub-phases: E2 concentration (pmol/L)^b^					
Follicular	Early	78	125	75.5 (18.4–78.5)	231 (192–283)
	Intermediate	83	172	95.6 (19.1–114)	294 (262–695)
	Late	84	464	182 (84–215)	858 (711–1337)
Ovulation		79	817	222 (98.5–283)	2212 (1598–3338)
Luteal	Early	85	390	188 (163–218)	658 (608–1394)
	Intermediate	81	505	244 (157–334)	1123 (942–1538)
	Late	84	396	111 (74.4–163)	815 (703–908)
Analysis of main menstrual cycle phases: LH concentration (IU/L)^c^					
Follicular		85	7.14	4.78 (3.17–5.04)	13.2 (12.4–17.8)
Ovulation		81	22.6	8.11 (6.37–10.1)	72.7 (67.4–100)
Luteal		85	6.24	2.73 (2.06–3.19)	13.1 (12.2–15.4)
Analysis of menstrual cycle sub-phases: LH concentration (IU/L)^c^					
Follicular	Early	78	6.41	3.12 (2.16–4.03)	9.79 (9.19–12.4)
	Intermediate	83	7.36	4.36 (3.01–4.59)	13.2 (12.5–15.6)
	Late	84	8.52	5.12 (3.89–5.58)	16.3 (15.2–26.5)
Ovulation		79	24.0	7.66 (5.10–9.40)	71.1 (65.4–100)
Luteal	Early	85	9.66	4.90 (1.96–4.98)	16.1 (15.1–30.2)
	Intermediate	81	5.36	1.97 (1.32–2.06)	11.6 (10.3–13.8)
	Late	84	5.00	1.86 (1.36–2.19)	11.7 (10.2–14.7)
Analysis of main menstrual cycle phases: progesterone concentration (nmol/L)^d^					
Follicular		85	0.212	0.159 (0.159–0.159)	0.616 (0.584–0.897)
Ovulation		81	1.81	0.175 (0.159–0.301)	13.2 (6.19–19.4)
Luteal		85	28.8	13.1 (8.34–15.6)	46.3 (43.2–64.8)
Analysis of menstrual cycle sub-phases: progesterone concentration (nmol/L)^d^					
Follicular	Early	78	0.380	0.159 (0.159–0.159)	1.03 (0.802–2.58)
	Intermediate	83	0.210	0.159 (0.159–0.159)	0.700 (0.619–3.44)
	Late	84	0.188	0.159 (0.159–0.159)	0.688 (0.579–12.3)
Ovulation		79	1.59	0.159 (0.159–0.171)	7.49 (5.91–19.4)
Luteal	Early	85	22.6	7.53 (4.66–9.53)	48.0 (39.9–54.1)
	Intermediate	81	39.2	15.2 (5.39–22.9)	66.5 (63.4–78.5)
	Late	84	18.2	1.71 (0.159–3.46)	43.1 (38.5–72.3)

CI, confidence interval; E2, estradiol; LH, luteinizing hormone.

a *N*, number of patients contributing to the data in this menstrual cycle phase (not number of samples).

b The measuring range for E2 is 18.4–11,010 pmol/L. Quantiles and CIs below the measuring range limits must be interpreted as ‘< measuring range’.

c The measuring range for LH is 0.3–200 IU/L. Quantiles and CIs below the measuring range limits must be interpreted as ‘< measuring range’.

d The measuring range for progesterone is 0.159–191 nmol/L. Quantiles and CIs below the measuring range limits must be interpreted as ‘< measuring range’.

### 3.3 Determination of E2, LH and progesterone values for menstrual cycle sub-phases

Analyses in menstrual cycle sub-phases showed that median E2 concentrations (Table 1; Fig. 1A) increased sharply from early (125 ​pmol/L) to late follicular (464 ​pmol/L) sub-phases, with a substantial peak during ovulation (817 ​pmol/L). Median E2 concentrations decreased sharply during the early luteal phase, but peaked again, to a lesser extent, in the intermediate luteal phase (505 ​pmol/L), before falling once again during the late luteal phase.

Median LH concentrations (Table 1; Fig. 1B) increased steadily from early follicular through to late follicular phases peaking during ovulation (24.0 IU/L), as observed in the initial three-phase analyses. During the ovulatory phase, LH showed the greatest IQR. Median LH concentrations fell steadily through early (9.66 IU/L) to late luteal (5.00 IU/L) sub-phases.

Median serum progesterone concentrations (Table 1; Fig. 1C) were low in the follicular phase and increased in the periovulatory phase, before increasing sharply to a peak level in the intermediate luteal phase (39.2 ​nmol/L), and then decreasing in the late luteal phase (18.2 ​nmol/L). The intermediate luteal phase showed the greatest IQR for progesterone.

## 4 Discussion

To date, this is the largest study to determine expected values for serum E2, LH and progesterone during each phase/sub-phase of the menstrual cycle, thereby establishing method-specific reference intervals for the Elecsys Estradiol III, LH and Progesterone III assays in normo-ovulatory women from the US and Europe. Knowledge of phase- and sub-phase-dependent fluctuations in these serum biomarkers could support informed clinical decision-making in women with hormone-related and fertility disorders. For example, early follicular phase E2 reference values are important for estimation of ovarian reserve (i.e. where anti-Müllerian hormone testing is not available) and for classification of anovulation, whereas mid-luteal progesterone values are important for confirmation of ovulation and adequate corpus luteum function. Importantly, provision of normal values for cycle sub-phases also permits accurate timing of ovulation for conception or contraception purposes [13].

Our results are consistent with previous studies describing serum concentrations of E2, LH and progesterone in normo-ovulatory women [14]. Studies conducted by Roos et al. [3] and Behre et al*.* [15] observed peaks in serum E2 and LH prior to ovulation and a rise in progesterone following ovulation; however, neither study established reference ranges for the individual sub-phases of the menstrual cycle, preventing direct comparison with the present analyses [3,15]. IQRs for E2, LH and progesterone levels in sub-phase analyses in our study were also consistent with previously documented variance in serum hormone profiles for natural menstrual cycles [[3], [4], [5]].

In the present study, serum LH values for menstrual cycle sub-phases provide further insight on LH fluctuations during the natural menstrual cycle. Serum E2, LH and progesterone concentrations were also in-line with hormone profiles previously characterized in urine, particularly with respect to the LH surge prior to ovulation [4]. Although urine and serum hormone profiles show good consistency, consensus has yet to be established on whether these may be used interchangeably [3,6]. Hormone profiles measured in serum are subject to swift metabolic clearance and pulsatile secretory input, whereas urinary profiles are subject to metabolism, and biomarker concentrations lag behind those observed in serum [6]. Equally, while serum hormone profiles may permit more accurate determination of hormones during the menstrual cycle phase and/or sub-phase, compared with those measured in urine, urine measurements may be more convenient for daily testing or at-home applications.

It is well documented that hormone profiles for E2, LH and progesterone fluctuate widely during the follicular and luteal phases of the natural menstrual cycle [1]. Serum E2, derived from a cohort of recruited small antral follicles, is low in the early follicular phase, but high in the late follicular phase when the selected pre-ovulatory follicle secretes high levels of the hormone [1]. Strengths of the current study include that it was a multicenter, international study, in which continuous sampling over the course of the natural menstrual cycle, at intervals of approximately 4 days, allowed for determination of high-resolution hormone profiles for one complete cycle, including sub-phases. Clinical interpretation of hormonal values should be based on sub-phase reference values to ensure accuracy, as assessment of ovarian reserve requires sampling of serum E2 (in addition to follicle-stimulating hormone) in the early follicular phase, and confirmation of ovulation with adequate corpus luteum function by measurement of progesterone in the mid-luteal phase. The use of sub-phase reference values for reproductive hormones in the follicular phase also aids informed clinical decision-making in assisted reproduction therapies, enabling timed intercourse or insemination through monitoring of the natural cycle.

Another strength of this study was that a large cohort was enrolled according to strict inclusion criteria, and expected values were determined in accordance with CLSI EP28-A3 guidelines. Study limitations include that self-reported health status was not validated by physical examination, and that there was no independent measurement of ovulation (i.e. using ultrasonography). Additionally, normalizing to a standard cycle length may also reduce the clinical utility of the findings where knowledge of the follicular phase data relative to cycle day and luteal phase data relative to number of days post-ovulation may be helpful**.** Finally, a lack of demographic diversity within the study population, where the majority of women enrolled were white/Caucasian, means that the present findings may not be applicable to all ethnic groups.

In conclusion, this study shows that intensive monitoring of the natural menstrual cycle by measurement of serum E2, LH and progesterone concentrations with the new generation Elecsys Estradiol III, LH and Progesterone III assays, allows reliable determination of these hormones and assignment to menstrual cycle phases. The expected values we have derived across all sub-phases of the menstrual cycle could assist informed clinical decision-making for women seeking to determine the day of ovulation (e.g. for timing of conception or contraception) or those with a range of fertility disorders.

## Declaration of competing interest

EA, AJ, JP, FR, MR, JS and MK report no conflict of interest. RP is an employee of Roche Diagnostics. KS is an employee of Roche Diagnostics GmbH and owns stocks/shares in Roche Diagnostics International Ltd.

## Data Availability

Data will be made available on request.
