# Supplementary material for: Extensive monitoring of the natural menstrual cycle using the serum biomarkers estradiol, luteinizing hormone and progesterone
Source: Pract Lab Med. 2021 Mar 13;25:e00211. doi: 10.1016/j.plabm.2021.e00211 (PMC8042396; doi:10.1016/j.plabm.2021.e00211)
Supplement: Multimedia component 1 [file mmc1.docx]

**SUPPLEMENTARY MATERIALS**

**Supplementary methods.** Including eligibility criteria, and additional methods for data collection, menstrual cycle standardization, sample processing and storage, determination of E2, LH and progesterone values and statistical analyses.

**Supplementary references.**

**Supplementary** **table S1.** Participant demographics.

**Supplementary table S2.** Standardization of cycle length**.**

**Supplementary table S3.** Standardization of menstrual cycle phases and sub-phases.

**Supplementary fig. S1.** Participant flow diagram.

**Supplementary methods**

*Additional eligibility criteria*

Additional inclusion criteria (detailed within the questionnaire) were a body mass index >19–<29 kg/m^2^ and agreement to follow the procedures documented within the protocol.

Exclusion criteria (detailed within the questionnaire) were: use of hormonal contraceptives (including morning-after pill) for at least 90 days prior to first blood draw and for the duration of the study; major surgery (using general anesthesia) up to 6 months prior to enrollment; fertility disorder; gonadal disorder/dysfunction; chronic endocrine disorder or dysfunction; chronic thyroid disorder/dysfunction; acute or chronic infections; human immunodeficiency virus, hepatitis C virus or hepatitis B virus infection; cardiac or vascular diseases; acute or chronic nephropathies; diabetes mellitus; hepatobiliary diseases; alcohol or drug abuse; previous or current cancer diagnosis or treatment; previous or current autoimmune diseases; use of investigational medicinal products within the past 90 days; and pregnancy or lactation.

*Data collection*

Relevant information on demographics and baseline characteristics, menstrual cycle, sample collection, specimen information, clinical information and adverse events was obtained for each participant and recorded via an electronic case report form.

*Menstrual cycle standardization*

Cycle phases (follicular/ovulation/luteal) were defined according to the day of ovulation, based on luteinizing hormone (LH) surge (if present) and/or estradiol (E2) and progesterone levels (Supplementary table S2).

Follicular and luteal phases were divided into three further sub-phases (early/intermediate/late), each with a length of 4.3 days (Supplementary table S3). The ovulation phase lasted 3 days.

*Sample processing and storage*

Freshly drawn samples were stored for 30–60 min at room temperature, and then processed to serum via centrifugation in accordance with the manufacturer’s instructions. Serum samples were stored in 0.4 mL aliquots at –80°C until measurement.

*Determination of E2, LH and progesterone values*

The Elecsys Estradiol III immunoassay uses a competition test principle, with a total duration of 18 minutes [1]. Coefficients of variation (CVs) for repeatability and intermediate precision (cobas e 411 analyzer) reported by the manufacturer in human serum pool (HSP) and control materials (PreciControl [PC] 1 and PC2) were 1.9–3.5% and 2.5–4.5%, respectively. The Elecsys LH immunoassay uses a sandwich test principle, with a total duration of 18 minutes [2]. CVs for repeatability and intermediate precision (cobas e 411 analyzer) reported by the manufacturer in HSP and control materials (PC1 and PC2) were 0.8–1.8% and 1.9–5.2%, respectively. The Elecsys progesterone III immunoassay uses a competition test principle, with a total duration of 18 minutes [3]. CVs for repeatability and intermediate precision (cobas e 411 analyzer) reported by the manufacturer in HSP and control materials (PC1 and PC2) were 1.2–11.8% and 3.6–23.1%, respectively.

Serum samples from eligible participants were randomized and tested in two batches at one central laboratory (UZ Brussels, Brussels, Belgium). Prior to analysis of any study samples, the central measuring site completed a familiarization experiment to confirm assay precision and reproducibility on control samples. Daily quality checks were conducted throughout the study to confirm measurement validity.

*Statistical analysis*

Estimated sample size for this study was calculated in accordance with Clinical and Laboratory Standards Institute (CLSI) EP28-A3c guidelines [4]. At least 59 evaluable participants were required for determination of reference ranges to assess the central 90th percentile; however, 120 are recommended. At least 120 evaluable participants were required for determination of the central 95th percentile. In cases where the sample size was <120, determination of expected values was deemed appropriate.

Serum E2, LH and progesterone were determined with the Elecsys Estradiol III, LH and Progesterone III assays, and captured electronically using an automated WinCAEv software. Statistical analyses and validation of expected values were conducted using R version 3.4.0 and SAS version 9.3 software, respectively.

Women with non-ovulatory menstrual cycles were excluded from the analysis based on a progesterone value of <8 ng/mL in the luteal phase. Other criteria for exclusion were: low LH in addition to very high progesterone levels during the luteal phase (suggestive of pregnancy or use of the morning-after pill); atypical profiles indicating a potential sampling error (e.g. high progesterone level within the follicular phase); study initiation not at the start of the menstrual cycle (e.g. high progesterone levels during the first phase of the cycle); or other anomalies (e.g. high progesterone levels throughout the cycle or low E2 values in the follicular phase).

Median values of E2, LH and progesterone were defined as the central 90% nonparametric range (quantiles 5% and 95%) determined for each parameter. Median values were reported, as opposed to mean values, to account for visit-participant correlation.

**Supplementary References**

1. Roche Diagnostics GmbH, Elecsys^®^ Estradiol III assay package insert (Method sheet). Mannheim, Germany, 2016.
2. Roche Diagnostics GmbH, Elecsys^®^ LH assay package insert (Method sheet), Mannheim, Germany, 2018.
3. Roche Diagnostics GmbH, Elecsys^®^ Progesterone III assay package insert (Method sheet). Mannheim, Germany, 2015.
4. Clinical and Laboratory Standards Institute (CLSI), EP28-A3c Defining, establishing, and verifying reference intervals in the clinical laboratory; approved guideline, 3rd ed., CLSI, Wayne, PA, 2010.

**Supplementary tables and figures**

**Supplementary** **table S1.**

Participant demographics.

|  | Evaluable participants (*N* = 85) |
| --- | --- |
| Age, years |  |
| Mean (±SD) | 26 (±4.43) |
| Median (Q1–Q3) | 25 (23–29) |
| BMI, kg/m^2^ |  |
| Mean (±SD) | 23.4 (±2.6) |
| Median (Q1–Q3) | 23 (21–25) |
| Height, cm |  |
| Mean (±SD) | 166 (±7.38) |
| Median (Q1–Q3) | 165 (160–172) |
| Weight, kg |  |
| Mean (±SD) | 64.3 (±8.6) |
| Median (Q1–Q3) | 65 (57.6–69.2) |
| Race, *n* (%) |  |
| White/Caucasian | 70 (82.4) |
| Black/African American | 7 (8.24) |
| Asian | 5 (5.88) |
| Other | 2 (2.35) |
| White/Caucasian Asian | 1 (1.18) |
| Ethnicity, *n* (%) |  |
| Not reported | 53 (62.4) |
| Not Hispanic or Latino | 31 (36.5) |
| Unknown | 1 (1.18) |
| Hispanic or Latino | 0 (0) |
| Work nightshift ≥3 times per week, *n* (%) |  |
| No | 79 (92.9) |
| Yes | 6 (7.06) |

BMI, body mass index; Q1, first quartile; Q3, third quartile; SD, standard deviation.

**Supplementary table S2**Standardization of cycle length**.**

| Phase^a^ | Standardization formula^b^ |
| --- | --- |
| Follicular | *Standardized day for the follicular phase = 1* $+\left( day-1 \right) x \frac{14}{length}$ |
| Luteal | *Standardized day for the luteal phase =* $15+\left( day-OV \right) x \frac{14}{length}$ |

Day, actual menstrual day; length, actual menstrual cycle phase length; OV, actual ovulation day.

^a^ Standardization of cycle length was performed separately for the follicular and luteal phases due to increased variation in follicular phase length.

**^b^** Cycle length and day of ovulation were standardized to account for variation in cycle length within the study population (24–35 days), and to enable determination of expected values for further sub-phases. The standardized cycle length was defined as 29 days, with day of ovulation occurring on menstrual cycle day 15. Measurements started between menstrual cycle days 1–3 due to individual enrollment into the study. Furthermore, sample collection exceeded 29 days in some participants as the last venipuncture did not coincide with the last menstrual cycle day.

**Supplementary table S3**Standardization of menstrual cycle phases and sub-phases.

| Phase^a^ | | Standardized days of cycle^b^ | |
| --- | --- | --- | --- |
|  |  | 3-phase | 7-phase |
| Follicular | Early | Day 1; ovulation –2 days | 0; 4.333 |
|  | Intermediate |  | 4.334; 8.667 |
|  | Late |  | 8.668; 13 |
| Ovulation | | Ovulation –1 day; ovulation +1 day | 14; 16 |
| Luteal | Early | Ovulation +2 days; last cycle day | 17; 20.333 |
|  | Intermediate |  | 20.334; 24.667 |
|  | Late |  | 24.668; 29 |

^a^ The time point of ovulation was determined individually for every subject. For this purpose, the luteinizing hormone (LH) maximum was identified per subject, using a standardized procedure based on a spline approach to model the LH values by day together with visual inspection. Parallel to the computations, all hormone profiles were examined by a medical expert. The ovulation time point calculated by the approach above was then compared with the thereby determined ovulation time point. In case of a questionable hormone profile, the ovulation day determined by the medical expert was applied. Based on pre-defined three cycle phases, the expected values per menstrual cycle phase were evaluated as the median parameter value.

^b^ See Supplementary table S2 for phase standardization formulas.


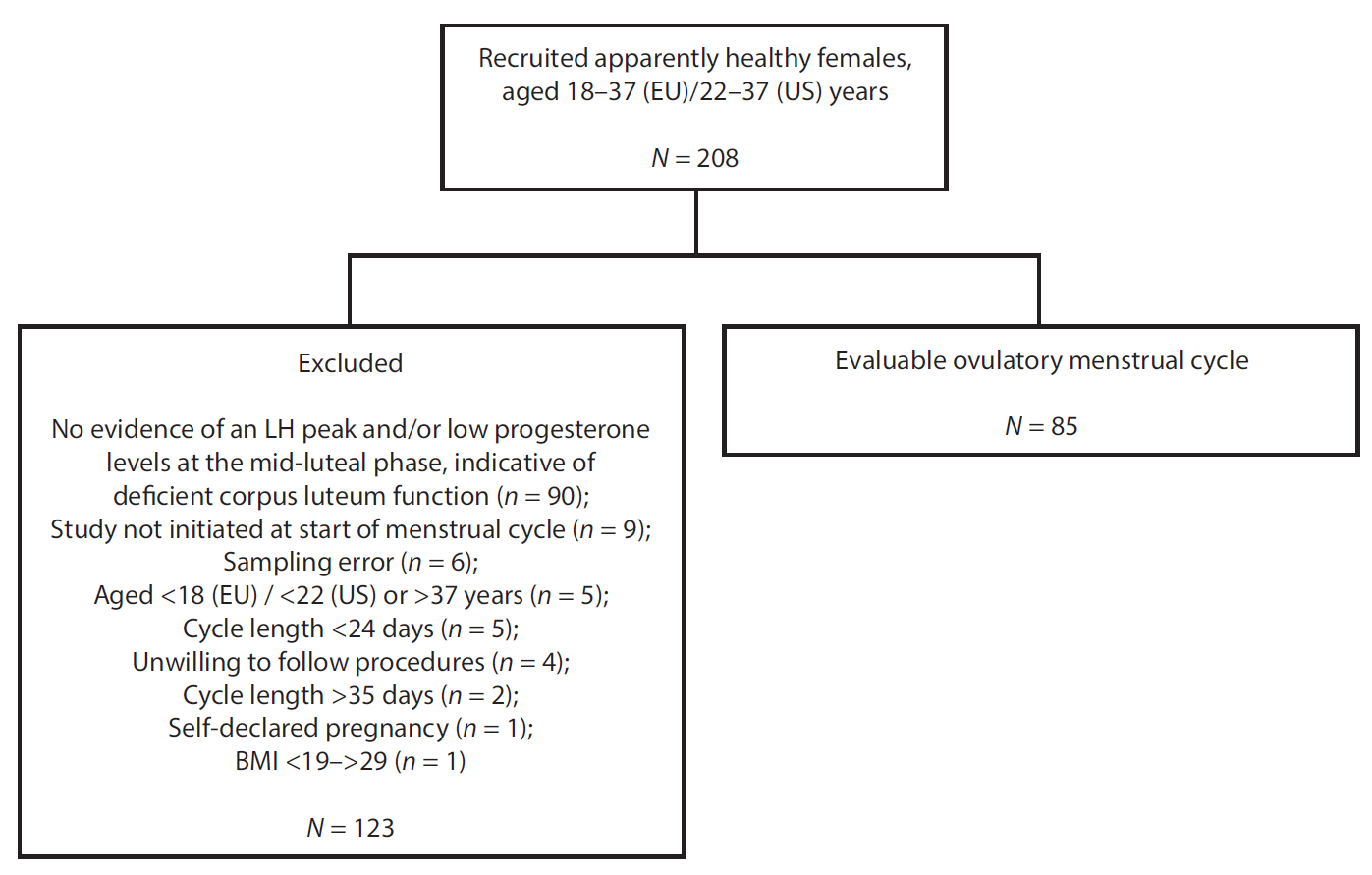
**Supplementary fig. S1.** Participant flow diagram.
BMI, body mass index; LH, luteinizing hormone; EU, European Union; US, United States of America.
